# LncMIR181A1HG is a novel chromatin-bound epigenetic suppressor of early stage osteogenic lineage commitment

**DOI:** 10.1038/s41598-022-11814-4

**Published:** 2022-05-11

**Authors:** Coralee E. Tye, Prachi N. Ghule, Jonathan A. R. Gordon, Fleur S. Kabala, Natalie A. Page, Michelle M. Falcone, Kirsten M. Tracy, Andre J. van Wijnen, Janet L. Stein, Jane B. Lian, Gary S. Stein

**Affiliations:** 1 Department of Biochemistry and University of Vermont Cancer Center, Larner College of Medicine at the University of Vermont, 89 Beaumont Avenue, Burlington, VT 05405 USA; 2 University of Vermont Cancer Center, University of Vermont Larner College of Medicine, Burlington, VT 05405 USA

**Keywords:** Cancer, Cell biology, Molecular biology

## Abstract

Bone formation requires osteogenic differentiation of multipotent mesenchymal stromal cells (MSCs) and lineage progression of committed osteoblast precursors. Osteogenic phenotype commitment is epigenetically controlled by genomic (chromatin) and non-genomic (non-coding RNA) mechanisms. Control of osteogenesis by long non-coding RNAs remains a largely unexplored molecular frontier. Here, we performed comprehensive transcriptome analysis at early stages of osteogenic cell fate determination in human MSCs, focusing on expression of lncRNAs. We identified a chromatin-bound lncRNA (MIR181A1HG) that is highly expressed in self-renewing MSCs. MIR181A1HG is down-regulated when MSCs become osteogenic lineage committed and is retained during adipogenic differentiation, suggesting lineage-related molecular functions. Consistent with a key role in human MSC proliferation and survival, we demonstrate that knockdown of MIR181A1HG in the absence of osteogenic stimuli impedes cell cycle progression. Loss of MIR181A1HG enhances differentiation into osteo-chondroprogenitors that produce multiple extracellular matrix proteins. RNA-seq analysis shows that loss of chromatin-bound MIR181A1HG alters expression and BMP2 responsiveness of skeletal gene networks (e.g., SOX5 and DLX5). We propose that MIR181A1HG is a novel epigenetic regulator of early stages of mesenchymal lineage commitment towards osteo-chondroprogenitors. This discovery permits consideration of MIR181A1HG and its associated regulatory pathways as targets for promoting new bone formation in skeletal disorders.

## Introduction

Skeletogenesis is mediated by multipotent mesenchymal stromal cells (MSCs) that have the ability to generate and regenerate bone tissues throughout the body. MSC-mediated osteogenesis is required during normal bone formation and remodeling processes, and is critically important during acute events such as tissue damage and fracture repair. The lineage commitment and progression of MSCs into osteogenic precursors, osteoblasts and mature osteocytes is a stringently controlled process that proceeds through several molecularly distinct stages^1,2^. MSCs are competent to self-renew, an important function necessary for clonal expansion to facilitate development and bone tissue repair, while maintaining progenitor pools for future utilization^3^. MSCs must tightly regulate cell cycle progression to prevent loss of self-renewal capacity. Beyond the importance of MSC function in physiological processes during development, the regulatory mechanisms of MSC fate determination are not fully understood. Because of the diverse biological roles for MSCs, they are controlled by a cascade of epigenetic regulators that provide a coordinated response to extrinsic and endocrine signals.

The commitment of MSCs to differentiated phenotypes in response to developmental cues is achieved by both chromatin-based and RNA-based mechanisms. While the mRNA expression patterns during osteogenesis have been extensively studied^4,5^, non-coding RNAs (e.g., microRNAs [miRNAs] and long non-coding RNAs [lncRNAs]) have been shown to be important in regulating the differentiation of osteoblasts^6^. LncRNAs are important factors that establish cell lineage commitment during tissue development with regulatory roles during adipogenesis^7^, keratinocyte differentiation^8^, and myogenesis^9^. Only a few studies have identified lncRNAs regulating MSC fate in the skeleton^10–14^, but in-depth characterization of the functional activities of lncRNAs is required to fully understand their proposed roles in regulating osteogenic cell fates.

Significantly, 75% of the genome encodes lncRNAs^15^, which are emerging in mammals as critical regulators of genomic structure through their diverse activities^16,17^. LncRNAs are generally expressed at lower levels than protein-coding transcripts, and their expression is often tissue specific. LncRNAs are multifunctional and can interact with numerous components of the gene regulatory machinery (proteins, RNA, DNA). LncRNAs function in a variety of biological processes, including sequestration (“sponging”) of miRNAs in the cytoplasm and regulating transcriptional activation or repression in the nucleus. DANCR^18^ and TUG1^19^ are examples of lncRNAs controlling proliferation and differentiation by regulating miRNAs that affect WNT signaling. LncRNAs are aberrantly expressed and represent potential biomarkers of osteoporosis, osteosarcoma, chrondrogenesis and osteoarthritis^20–23^. LncRNAs are functionally linked to bone abnormalities. For example, Hotair disruption results in malformation of metacarpal-carpal bones and homeotic transformation of the spine^24^. Knockout mice of the miR-host-Dnm3os lncRNA die shortly after birth and exhibit skeletal abnormalities, including craniofacial hypoplasia, vertebral defects, and osteopenia^25^. LncRNA HULC promotes progression of bone neoplasms^26^. Although these studies point to the importance of lncRNAs in regulating bone homeostasis through regulation of osteogenesis, there is a need to expand identification and characterization of lncRNAs that support the commitment of MSCs to osteoblasts.

Here, we identify lncRNA MIR181A1HG as a differentially expressed lncRNA that is down-regulated during early stages of MSC commitment into osteo-chondrogenic progenitor cells. We establish a novel function for MIR181A1HG in the stabilization of the uncommitted MSC phenotype and in preventing DNA damage during self-renewal in uninduced MSCs. Knockdown of MIR181A1HG at the onset of BMP2-induced osteogenic lineage commitment increases expression of the osteogenic marker alkaline phosphatase (ALP; gene symbol ALPL) and a number of extracellular matrix proteins. Transcription factor SOX5, a regulator of osteochondral differentiation^27^, is downregulated upon MIR181A1HG knockdown, thereby resulting in altered expression of its downstream targets. These findings identify a novel lncRNA for further characterization as a therapeutic intervention for skeletal disorders.

## Results and discussion

### lncRNA MIR181A1HG is expressed in MSCs and other human tissues

To determine which lncRNAs are differentially expressed during lineage commitment, we performed transcriptome profiling of TERT-immortalized human mesenchymal stromal cells (hTERT20-hMSC) in osteogenic media (containing BMP2, dexamethasone, ascorbic acid and β-glycerophosphate). Three time points corresponding to distinct phases of mesenchymal lineage commitment and progression—proliferation (Day 0), commitment (Day 7) and extracellular matrix production (Day 14) were used to evaluate gene expression. Analysis of RNA-sSeq revealed that there are 15,768 mRNAs and 9,173 lncRNAs expressed over the 3 temporal phases of differentiation. Expression of established mRNA biomarkers for cell proliferation, differentiation and/or function revealed that these cells transition into immature osteochondrogenic progenitors that express select osteo-, chondro- and adipogenic markers (Supplementary Fig. 1). Hence, the differentiation time course of TERT-MSCs under our conditions resembles a model for early stages of osteochondrogenic differentiation.

**Figure 1:**
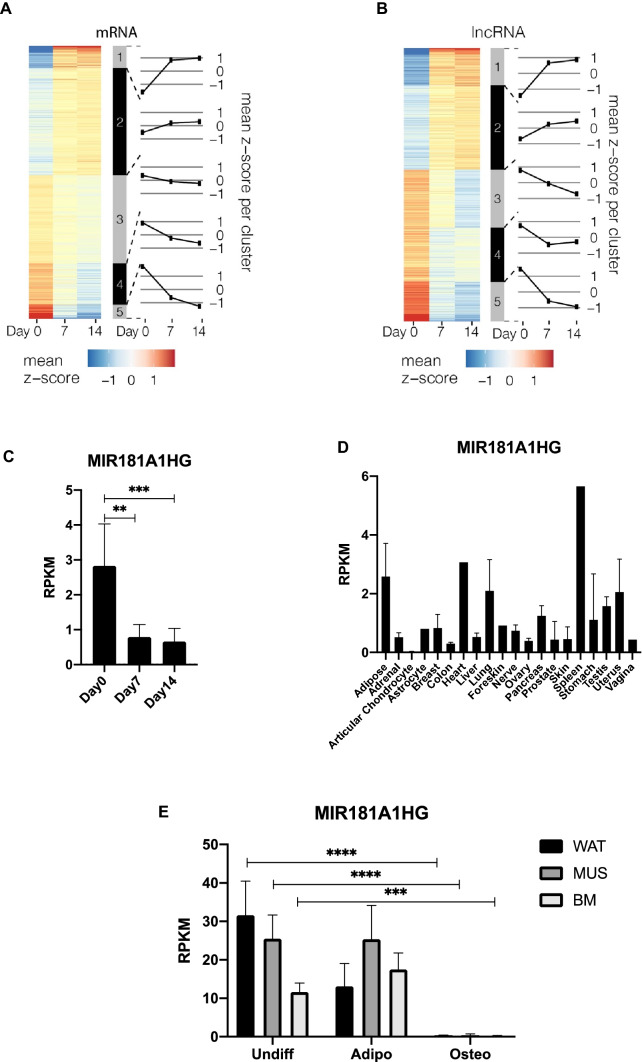
MIR181A1HG expression decreases with osteogenic differentiation. (**A**) Hierarchical clustering of 6185 DE mRNAs in hMSC-hTERT20 cells. (**B**) Hierarchical clustering of 1153 DE lncRNAs in the same hMSC-hTERT20 (n = 6). (**C**) MIR181A1HG expression decreases with osteogenic differentiation of tert-immortalized hMSCs (hMSC-hTERT20) (n = 6). ** padj < 10^–4^; *** padj < 10^–7^. (**D**) MIR181A1HG expression across human tissues (n = 1–2). The lncRNA is ubiquitously expressed across all examined cells and tissues except in articular chondrocytes. Datasets from twenty-one adult human tissues were downloaded from the ENCODE project^50^ and examined for MIR181A1HG expression. (**E**) MIR181A1HG expression in MSCs derived from white adipose tissue (WAT), muscle (MUS) and bone marrow (BM) undergoing adipogenic and osteogenic differentiation for 9 days^28^. MIR181A1HG expression is significantly decreased after osteogenic differentiation compared to mulitpotent or adipogenic cells (n = 3). ***padj < 10^–7^, ****padj < 10^–10^. Data are presented as mean ± SD.

Unbiased differential expression (DE) analysis revealed 6,185 mRNAs (Fig. 1A) and 1,153 lncRNAs (Fig. 1B) with an absolute fold-change > 1.5 across the time-course. Clustering of the differentially expressed genes showed distinct profiles at each time point corresponding to the patterns of relative increase (or decrease). Two clusters were associated with temporal increases in gene expression (Fig. 1A,B: clusters 1–2) while the remaining 3 clusters (Fig. 1A,B: clusters 3–5) demonstrated a relative decrease in expression during the time-points tested. Comparison of the mRNA and lncRNA expression profiles (Fig. 1A,B) suggest that genes with the same temporal expression patterns have related biological roles in regulating commitment (day 7) and progression (day 14) to osteochondrogenesis.

We focused our analysis on MIR181A1HG, because this lncRNA has not previously been associated with osteogenesis. This lncRNA is highly expressed in proliferating hMSC-hTERT20 cells and its expression decreases during differentiation (Fig. 1C). MIR181A1HG expression is also evident and variable in other human cell types and tissues (Fig. 1D), indicating that this lncRNA has broader cell lineage-related functions. Next, we examined expression of MIR181A1HG after adipogenic and osteogenic differentiation of MSCs isolated from three sources: white adipose tissue (WAT), muscle (MUS) and bone marrow (BM)^28^. MSCs from all three tissues expressed MIR181A1HG in the undifferentiated state as well as after 9-days of adipogenic differentiation (Fig. 1E). However, MIR181A1HG expression was significantly decreased after osteogenic differentiation of all three subtypes of MSCs (Fig. 1E). This finding supports the hypothesis that MIR181A1HG represents a novel regulator of osteochondral lineage commitment during skeletal development and mineralized tissue formation.

### MIR181A1HG is a nuclear lncRNA that supports cell cycle progression and maintains DNA integrity in MSCs

**Figure 2:**
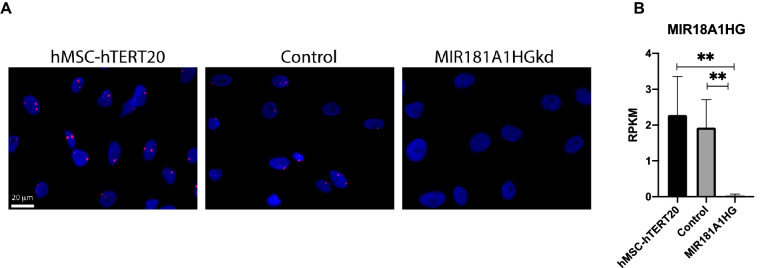
MIR181A1HG is a nuclear lncRNA expressed highly in proliferating MSCs and expression is depleted by CRISPRi. (**A**) Representative RNA-FISH images of MIR181A1HG in hMSC-hTERT20 (left), CRISPRi control (middle) and MIR181A1HGkd (right) cells. Red spots indicate expressed RNA. Nuclei are counterstained with DAPI. Scale bars represent 20 µm. (**B**) RNA transcript levels in reads per kilobase million (RPKM) of MIR181A1HG in parental hMSC-hTERT20 (n = 6), CRISPRi control and MIR181A1HGkd cells (n = 3), as assessed by RNA-Seq. **padj < 10^–4^. Data are presented as mean ± SD.

To gain insight into the functions of the lncMIR181A1HG, we examined MSCs maintained under conditions that support growth in order to evaluate effects on self-renewal. Subcellular localization by RNA fluorescent in situ hybridization (RNA-FISH) with proliferating hMSC-hTERT20 cells revealed that MIR181A1HG was primarily nuclear and essentially undetectable in the cytoplasm of MSCs (Fig. 2A left).

To evaluate determine the regulatory activity of MIR181A1HG, we knocked down its expression in the hMSC-hTERT20 cell line by CRISPR interference (CRISPRi; MIR181A1HGkd). Transcriptome analysis confirmed that CRISPRi achieved greater than 95% knockdown of MIR181A1HG expression compared to hMSC-hTERT20 and CRISPRi control cells (Fig. 2B). This knockdown was verified by the absence of detectable MIR181A1HG transcript by RNA-FISH in the MIR181A1HGkd CRISPR-modified cells (Fig. 2A right) and presence of MIR181A1HG RNA in the control cells (Fig. 2A center).

**Figure 3:**
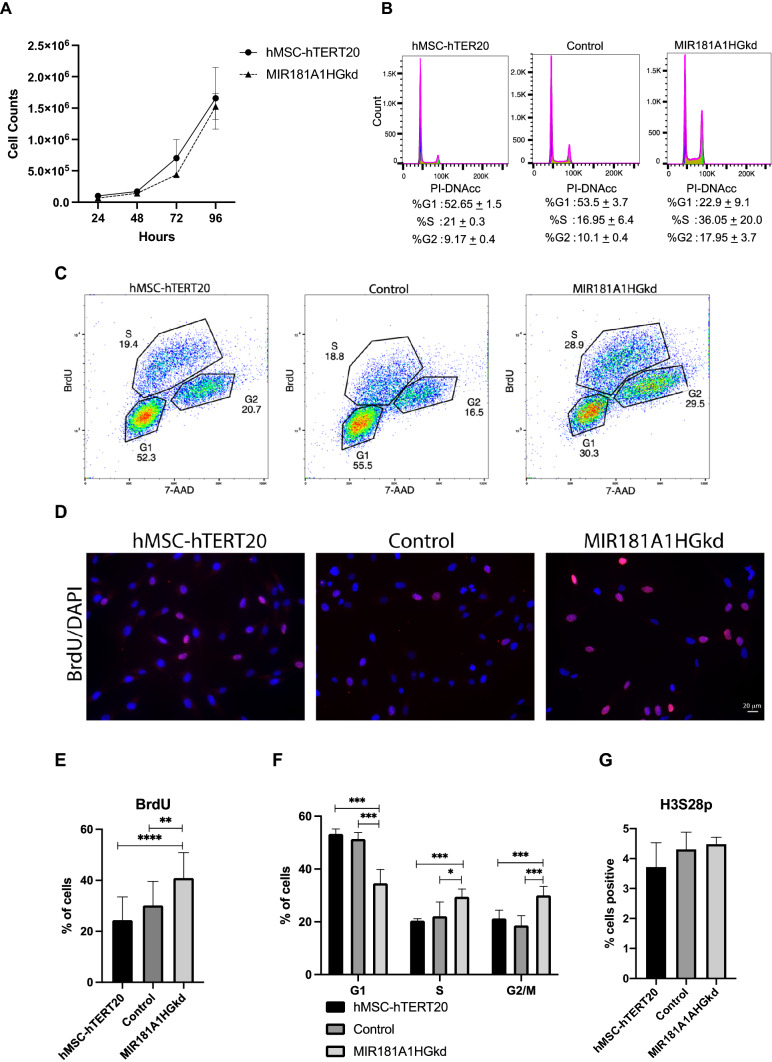
MIR181A1HGkd causes cell cycle progression defects. (**A**) Proliferation curves of hMSC-hTERT20 and MIR181A1HGkd cells. (**B**) Cell-cycle distribution of Control and MIR181A1HG-depleted cells by FACS with PI staining (n = 2). (**C**) Cells were stained for BrdU incorporation and total DNA content by 7-AAD for FACS analysis. Representative cell-cycle distribution of hMSC-hTERT20, CRISPRi control and MIR181A1HG-depleted cells. (**D**) Representative images of BrdU immunofluorescence after 30 min incorporation. BrdU incorporation is red, while nuclei are counterstained with DAPI (blue). Scale bars represent 20 µm. (**E**) Quantification of BrdU incorporation by immunofluorescence (n > 700cells). **p < 0.01; ****p < 0.0001. (**F**) Quantification of BrdU cell cycle profiling of hMSC-hTERT20, control and MIR181A1HGkd cells. * p < 0.05; **p < 0.01; ***p < 0.005 (n = 5). (**G**) No significant difference was observed in phosphorylation of the mitotic marker H3S28 with MIR18A1HGkd (n > 700 cells). Data are presented as mean ± SD.

Depletion of MIR181A1HG had no effect on overall cell proliferation of the MSCs as evidenced by growth rate (Fig. 3A). However, changes in cell cycle profiles were evident upon MIR181A1HG knockdown. Total DNA content was examined using Fluorescence Activated Cell Sorting (FACS) analysis with propidium iodide (PI) labeled cells. Compared to controls, MIR181A1HG-depleted cells had an increase in the proportion of cells in S phase and G2/M phases, with a commensurate reduction in the G1 phase population (Fig. 3B,C). These flow cytometry data indicate a delay in cell cycle progression after the onset of DNA replication at the G1/S phase transition. Analysis of BrdU incorporation by immunofluorescence microscopy also showed an increase in S phase (Fig. 3D, E). FACS analysis of BrdU incorporation confirmed the increase in both S and G2/M seen with PI staining (Fig. 3F). Due to the effect of MIR181A1HG knockdown on G2/M phases, we used the mitotic marker histone H3 serine 28 phosphorylation (H3S28p) to assess if it was contributing to the higher G2/M population. No significant increase in the mitotic marker H3S28p was observed in the MIR181A1HGkd cells (Fig. 3G). Hence, loss of MIR181A1HG affects cell cycle progression.

**Figure 4:**
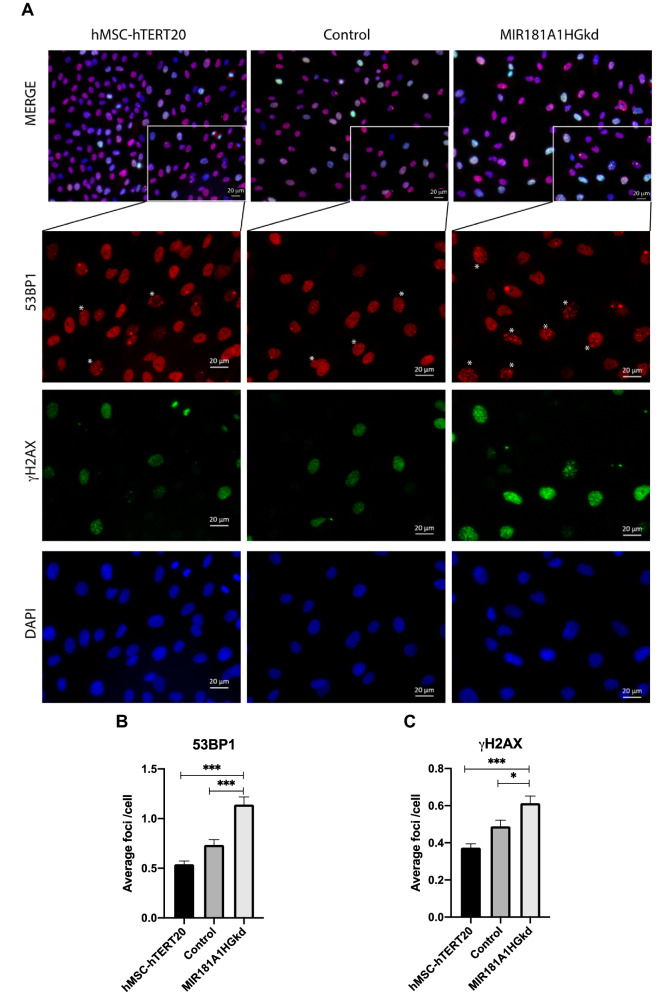
MIR181A1HGkd cells show increased DNA damage as assessed by 53BP1 and γH2AX staining. (**A**) Immunofluorescence microscopy of hMSC-hTERT20 cells, CRISPRi control and MIR181A1HGkd for 53BP1 (red) and γH2AX (green). Nuclei were counterstained with DAPI (blue). 53BP1 and γH2AX are diffuse in the nucleoplasm in undamaged cells and upon damage/dsDNA breaks, shows punctate focal staining patterns. Cells with high γH2AX foci are highlighted by *. Scale bars represent 20 µm. Quantification of the number of (**B**) γH2AX and (**C**) 53BP1 foci per nuclei was made using Volocity. For each cell line, > 700 cells were analyzed from 2 independent experiments. *p < 0.05; ***p < 0.005. Data are presented as mean ± SD.

We then evaluated if MIR181A1HG loss of function compromises DNA integrity. We performed immunostaining for γH2AX, the phosphorylated form of histone H2AX that marks double-strand breaks (DSB)^29^, and 53BP1 which is a crucial component of DNA DSB signaling and repair^30^. MIR181A1HG knockdown cells showed increases in both γH2AX and 53BP1 foci compared with control cells, indicative of increased DSBs (Fig. 4). Because inhibition of MIR181A1HG increases DNA damage during interphase, it appears that MIR181A1HG function is required for normal cell cycle progression, because it maintains chromosomal integrity and MSC phenotype stability in the S and G2 phases during self-renewal of MSCs.

### MIR181A1HG knockdown induces osteogenic differentiation and increases extracellular matrix gene expression

**Figure 5:**
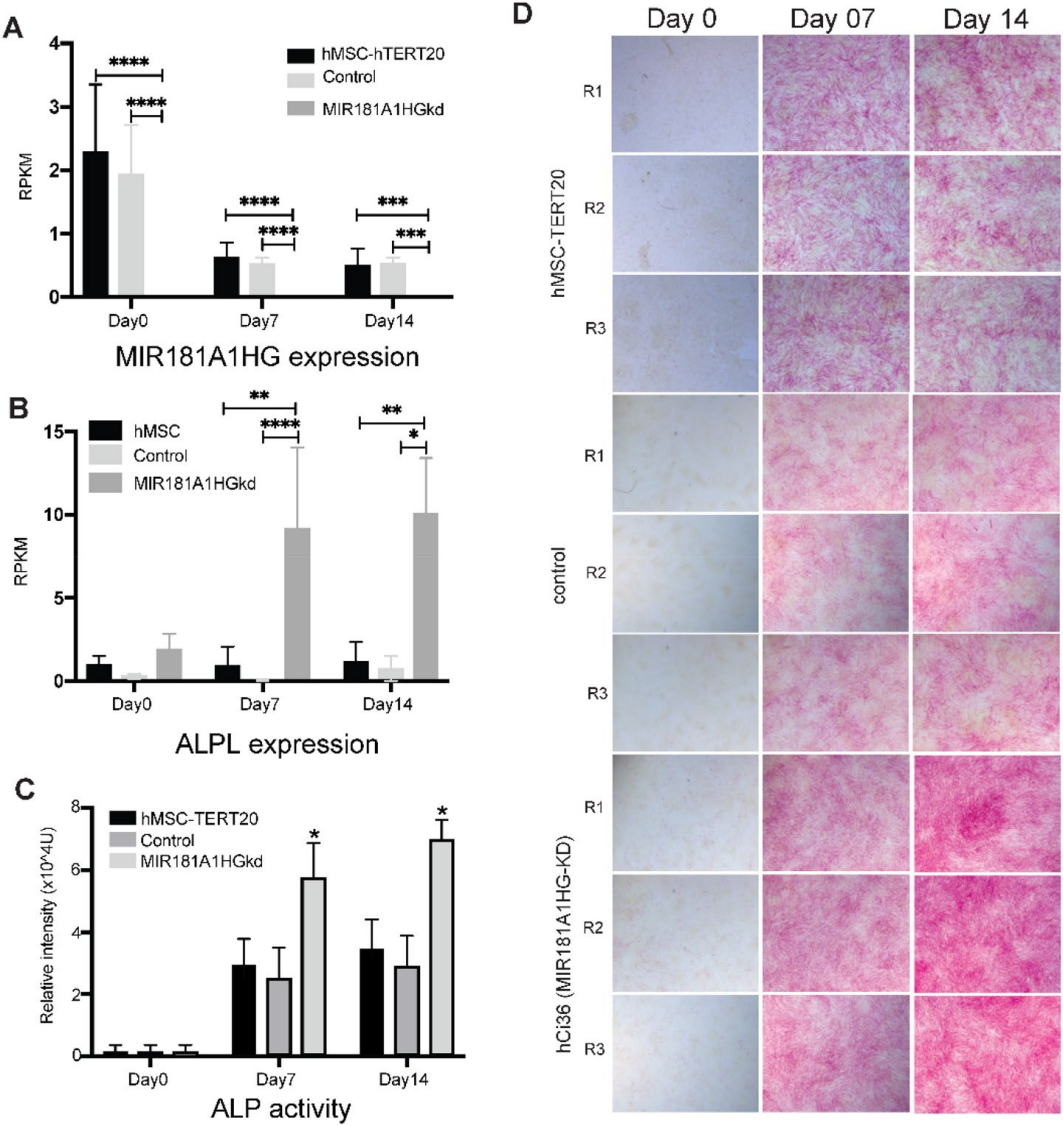
MIR181A1HG knockdown increases ALP expression and activity after osteogenic differentiation. MIR181A1HG was depleted by CRISPRi using dCas9-KRAB and cells were induced to osteogenic differentiation for 14 days. (**A**) MIR181A1HG expression in hMSC-hTERT20, CRISPRi control and MIR181A1HGkd cells by RNA-Seq. (**B**) RNA expression of ALPL from global transcriptome analysis. (**C**) Quantitation of alkaline phosphatase staining from (**D**). (**D**) Cells were stained for ALP activity; MIR181A1HGkd cells showed increased staining at days 7 and 14 compared to parental hMSC-hTERT cells and CRIPSRi controls. *padj < 0.05; **padj < 0.01; ***padj < 0.001; *****padj < 10^–4^ (n = 3). Data are presented as mean ± SD.

Because skeletal development involves both osteogenic and chondrogenic contributions to bone formation, we further evaluated expression of genes associated with both. We addressed the hypothesis that MIR181A1HG is expressed in multipotent MSCs and functions as an inhibitor of osteogenesis and maturation of mineralized tissue. We therefore examined how MIR181A1HG knockdown influences osteogenic differentiation of hMSC-hTERT20 cells at days 0, 7 and 14. The CRISPRi-modified cells sustained repression of MIR181A1HG throughout the differentiation time-course, and the CRISPRi control cell line showed expression comparable to the parental hMSC-hTERT20 cells (Fig. 5A). We assayed ALPL RNA expression and found that it was strikingly increased at both days 7 and 14 compared to the control and hMSCs (Fig. 5B). Cells were then stained for alkaline phosphatase activity (ALP), a marker of osteogenesis. At day 0 before differentiation was initiated, each of the images showed absence of ALP staining. Both the CRISPRi control cells and the hMSC-hTERT20 cells at days 7 and 14 showed ALP staining but without changes between days 7 and 14 (Fig. 5C and D). Importantly, day 14 shows an increase in ALP staining with MIR181A1HG knockdown compared to day 7 (Fig. 5C and D). Thus, the knockdown of MIR181A1HG promotes upregulation of alkaline phosphatase at both the RNA and protein levels, consistent with initiation of osteoblastogenesis.

With the increased ALP activity following MIR181A1HG knockdown, we addressed the alterations in gene expression upon MIR181A1HG depletion. Transcriptome changes were compared to CRISPRi control cells at three differentiation time points to determine the effect of MIR181A1HG knockdown on osteogenesis. A total of 1847 genes exhibited greater than twofold difference in expression compared to control. Of these, 1580 were mRNAs and 267 were lncRNAs.

LncRNAs can regulate gene expression through several mechanisms, functioning either in cis or trans^31^. To determine if MIR181A1HG regulates genes in cis or trans, we performed positional gene enrichment (PGE)^32^ on the genes that were differentially expressed after knockdown. MIR181A1HG is located on chromosome 1q32. Regions cis to MIR181A1HG show significant enrichment of DE genes, suggesting some cis regulation (Supplementary Fig. 2). The most significant enrichment of differentially expressed genes was on chromosomes 3, 5, 11, 12 and 18, indicating that MIR181A1HG also affects genes in trans.

**Figure 6:**
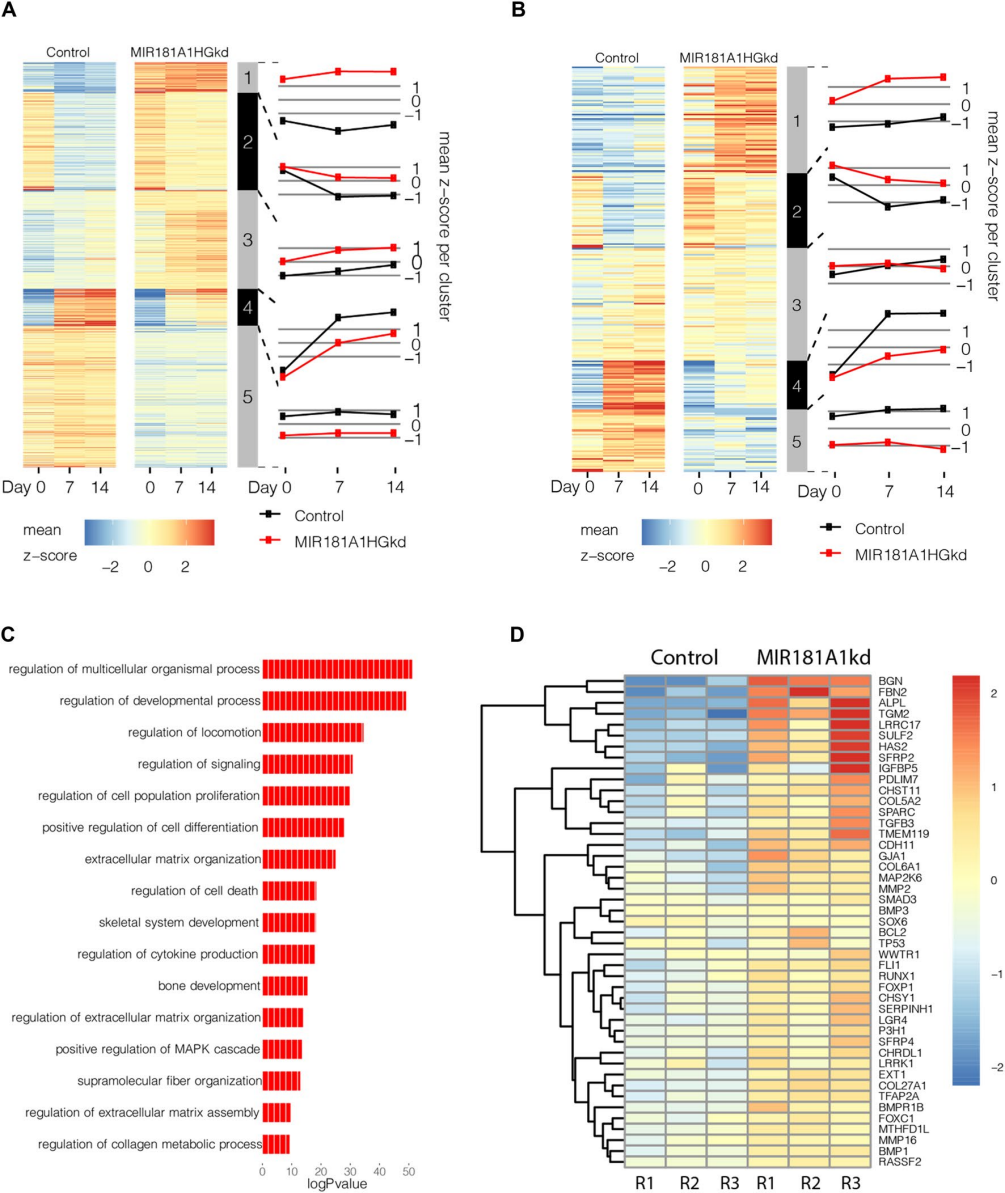

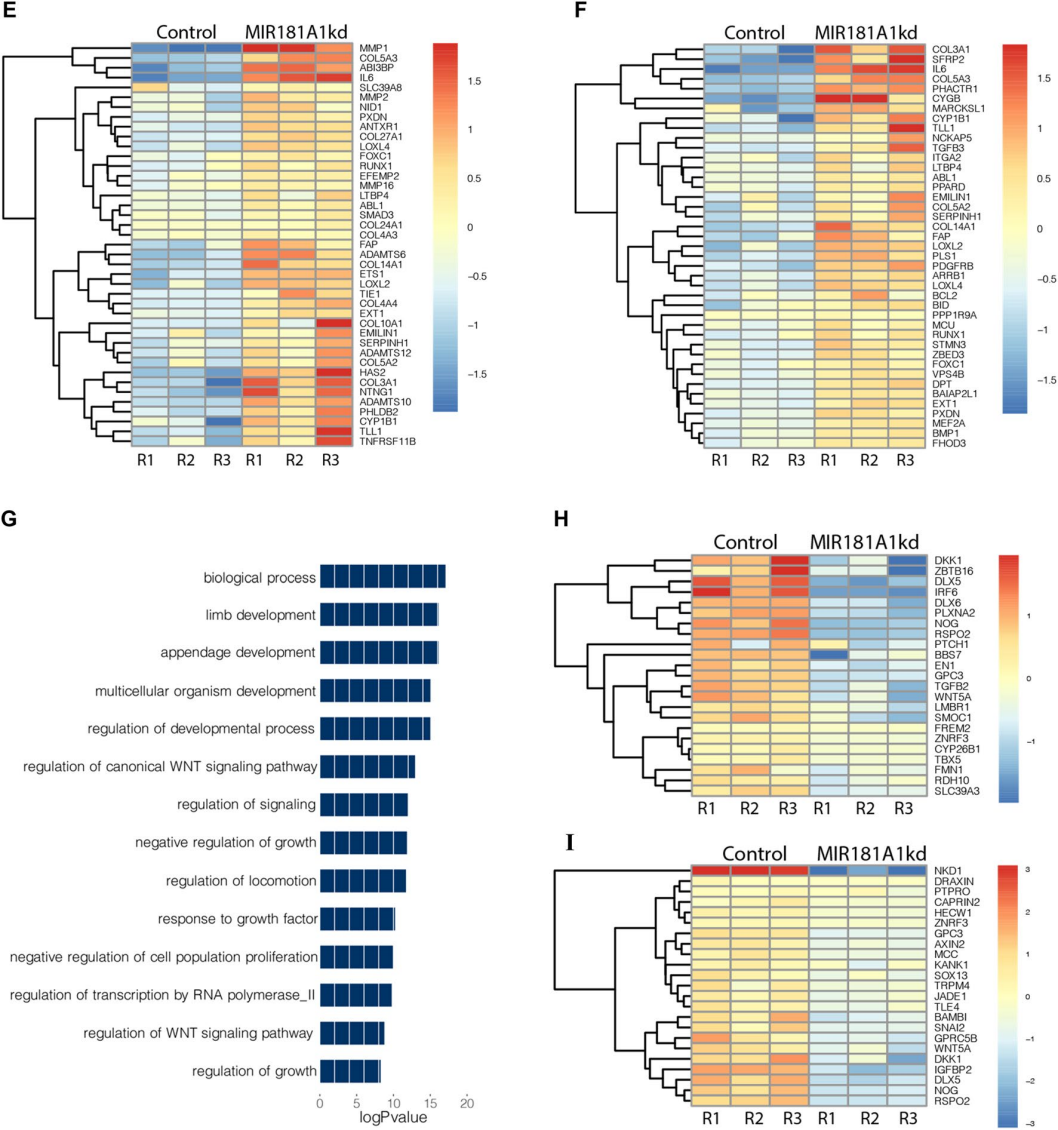
MIR181A1HG knockdown causes an increase in extracellular matrix gene expression. (**A**) Expression profile of 1580 DE mRNAs in CRISPRi modified hMSC-hTERT20 cells (red) compared to CRISPRi control cells (black) (n = 3). (**B**) Expression profile of 267 DE lncRNAs in the same MIR181A1HGkd and control cells. (**C**) Gene Ontology of mRNAs with increased expression after MIR181A1HGkd at Day 7. (**D**) Upregulated genes at day7 associated with skeletal and bone development. (**E**) Upregulated genes at day7 associated with extracellular matrix organization. (**F**) Upregulated genes at commitment associated with collagen and supramolecular fiber organization. (**G**) Gene Ontology of mRNAs decreased expression after MIR181A1HGkd Day7. (**H**) Gene Ontology of downregulated genes at commitment associated with limb and appendage development. (**I**) Gene Ontology of downregulated genes at commitment associated with WNT signaling.

Hierarchical clustering of the differentially expressed mRNAs (Fig. 6A) and lncRNAs (Fig. 6B) showed distinct profiles across the differentiation time-course with MIR181A1HG knockdown. Clusters 1–2 show both the mRNAs and lncRNAs having the highest levels as a result of the lncRNA knockdown, while Clusters 4–5 show higher levels in the control than in the knockdown. Examination of the 884 protein-coding genes that were expressed at higher levels with MIR181A1HG knockdown (Clusters 1–3) identified genes associated with collagen fibril and extracellular matrix (ECM) organization; bone development and ossification; regulation of ERK and MAPK signaling; regulation of cell proliferation, regulation of cell death (Supplementary Fig. 3). Clusters 4 and 5 depict the 696 mRNAs that were generally lower in expression after MIR181A1HGkd and included genes associated with negative regulation of cell migration, limb development, regulation of cytosolic calcium ion concentration and negative regulation of growth (Supplementary Fig. 4).

Focusing on the overall role of MIR181A1HG in MSC commitment to osteochondrogenesis, we further examined the effect on gene expression at Day 7. At this time point, we identified 745 mRNAs that were upregulated upon MIR181A1HG knockdown. As expected, gene ontology was aligned with global changes associated with osteogenesis and chondrogenesis (Supplementary Fig. 3). Expression of several genes were associated with extracellular matrix organization, skeletal system development and bone development (Fig. 6C). We had previously observed an increase in ALP activity with differentiation by histochemistry (Fig. 5B) and this was supported by the increase in ALP expression at the gene level. Many other genes related to bone development were also upregulated with MIR181A1HG knockdown (Fig. 6D).

With differentiation experiments in culture, the MIR181A1HG knockdown resulted in an increase in extracellular matrix and/or collagen production. When isolating the MIR181A1HGkd samples at days 7 and 14 of differentiation, an increase of extracellular matrix/collagenous material was consistently observed (data not shown). This observation is supported by the increased expression of collagen and ECM associated genes seen with MIR181A1HGkd at commitment (day 7) (Fig. 6E,F).

Examination of the downregulated genes with MIR181A1HG knockdown at commitment identified 662 mRNAs (Fig. 6G). The ontological associations with the downregulated genes were not as significant; however some categories with osteogenic implications included limb and appendage development (Fig. 6H) and regulation of WNT signaling (Fig. 6I), which contribute to osteoblast differentiation. The downregulated mRNAs were largely associated with general categories, i.e., biological process, multicellular organism development and developmental regulatory processes (Fig. 6G). Within these broader gene ontology categories, we noted that MIR18A1HG knockdown also specifically suppressed the induction of a number of BMP2 responsive genes that normally attenuate BMP signaling (e.g., NOG/noggin, SMAD6, SMAD9, ADAM19). This finding suggests that MIR18A1HG is normally required to support negative feedback regulation during BMP2 signaling in MSCs.

These loss-of-function effects of MIR181A1HG collectively reinforce the concept that this lncRNA contributes at a decisive point for phenotype commitment during early stages of BMP2-induced cell fate determination in the osteochondroblast lineage.

### MIR181A1HG is a nuclear lncRNA that interacts with chromatin, affecting expression of the transcription factor SOX5 and its downstream targets

**Figure 7:**
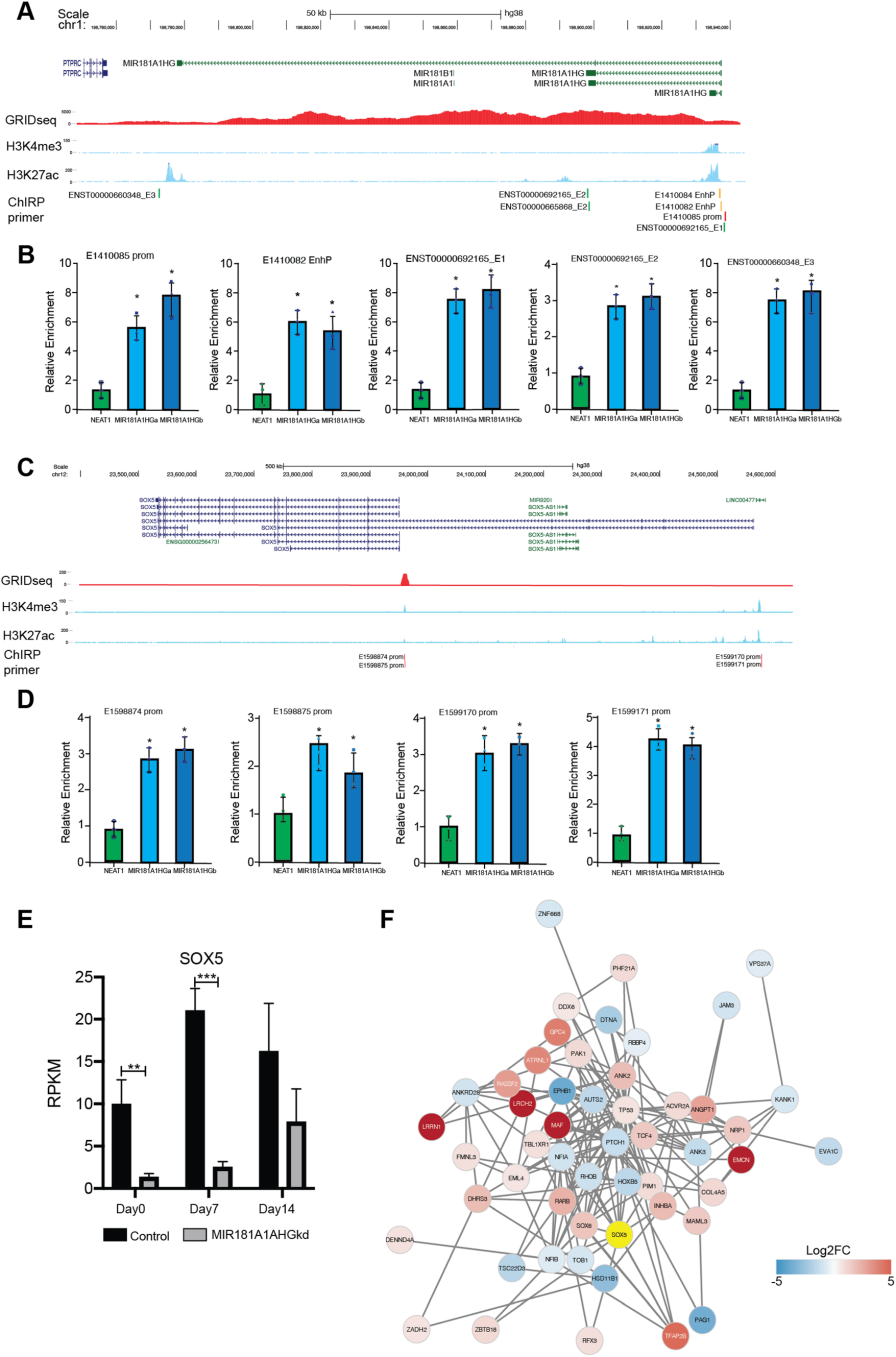
MIR181A1HG Interacts with Chromatin and Regulates SOX5 expression and its downstream targets. (**A**) UCSC genome browser ideogram depicting MIR181A1HG locus on chromosome 1 with associated regulatory regions depicted. GRIDseq data from MDA-MB-231 cells show occupancy of MIR181A1HG on chromosome 1 (red). Specific histone modification (H3K4me3 and H3K27ac) ChIPseq tracks from MSCs are depicted, demonstrating active regulatory regions around assigned gene promoters (blue). (**B**) Quantitative PCR of ChIRP DNA from MIR181A1HG-associated probes. For all genomic regions tested MIR181A1HG demonstrated association with specified genomic regions for both probe sets used (MIR181A1HGa and MIR181A1HGb). Relative enrichment compared to control LINC (NEAT1) demonstrated that MIR181A1HG is specifically associated with the MIR181A1HG gene locus and regulatory regions. Statistical significance was assessed using one-way ANOVA followed by Bonferroni post-test (*p < 0.05). Data are presented as mean ± SD. (**C**) UCSC genome browser ideogram depicting SOX5 locus on chromosome 12 with associated regulatory regions depicted. GRIDseq data from MDA-MB-231cells show occupancy of MIR181A1HG on chromosome 12 near the SOX5 promoter region (red). Specific histone modification (H3K4me3 and H3K27ac) ChIPseq tracks from MSCs are depicted, demonstrating active regulatory regions around assigned gene promoters. (**D**) Quantitative PCR of ChIRP DNA from MIR181A1HG-associated probes. For all genomic regions tested MIR181A1HG demonstrated association with specified genomic regions for both probe sets used (MIR181A1HGa and MIR181A1HGb). Relative enrichment compared to control LINC (NEAT1) demonstrated that MIR181A1HG is specifically associated with the SOX5 gene regulatory regions. Statistical significance was assessed using one-way ANOVA followed by Bonferroni post-test (*p < 0.05). (**E**) SOX5 is downregulated with MIR181A1HG knockdown cells. * padj < 0.001; **padj < 10^–4^; ***padj < 10^–7^ (n = 3). Data are presented as mean ± SD. (**F**) STRING network of differentially expressed SOX5 targets from curated TRANSFAC database. DE mRNAs are shown with log2FC scale. SOX5 is highlighted in yellow^55^.

Having determined that MIR181A1HG is a nuclear RNA (Fig. 2A), we examined if this lncRNA binds to chromatin. Examination of publicly available GRID-Seq (global RNA interactions with DNA by deep sequencing) data from a different cell line, MDA-MB-231 cells^33^, identified MIR181A1HG as binding to chromatin both in cis, where it is expressed on chromosome 1, and in trans at the SOX5 gene on chromosome 12 (Fig. 7A and C). We examined the expression of SOX5 and observed that knockdown of MIR181A1HG in our hMSC-hTERT20 cells causes decreased expression of SOX5 (Fig. 7E). SOX5 is a known inhibitor of osteogenesis, and its knockdown has been demonstrated to increase ALP activity and expression of osteoblast markers including collagen^34^.

We then performed Chromatin Isolation by RNA Purification (ChIRP) experiments and established that the lncRNA MIR181A1HG binds to both its own promoter and to SOX5 in MSCs (Fig. 7). Specific histone modifications (H3K4me3 and H3K27ac) based on ChIPseq tracks from MSCs demonstrate active regulatory regions associated with both the MIR181A1HG (Fig. 7A) and SOX5 (Fig. 7C) loci. For specific genomic regions tested, lncRNA MIR181A1HG showed association for both probe sets used (MIR181A1HGa and MIR181A1HGb). Relative enrichment compared to the control LINC (NEAT1) revealed that lncRNA MIR181A1HG is bound to both the MIR181A1HG (Fig. 7B) and the SOX5 (Fig. 7D) gene loci and regulatory regions. These data support that MIR181A1HG specifically binds and potentially regulates SOX5, similar to GRID-seq findings in MDA-MB-231. Together these findings support an essential chromatin interaction that is important for maintaining the osteo-chondroprogenitor function of MSCs.

We compiled a list of 258 genes known to be regulated by SOX5 from the TRANSFAC Curated Transcription Factor Targets dataset^35^. These genes were compared to our MIR181A1HGkd transcriptome and 52 were found to be differentially expressed with MIR181A1HG knockdown (Fig. 7F). Examining a selection of the differentially expressed genes that are downstream of SOX5, we found Patched 1 (PTCH1) is decreased with MIR181A1HGkd. PTCH1 is a known regulator of osteogenesis^36^ and increased bone mass has been observed in PTCH1-deficient mice and patients. Additionally, PTCH1-deficient osteoblast precursor cells have been shown to differentiate at an accelerated rate, which is consistent with the observed MIR181A1HGkd phenotype (Fig. 5). Similarly, nuclear factor 1 A (NFIA) is downregulated with MIR181A1HGkd and NFIA knockdown has been shown to promote osteoblast differentiation^37^. Downstream gene Ankyrin 2 (ANK2) expression increases with MIR181A1HGkd and ANK2 is a key regulator of bone mineralization^38^. TCF4 is another gene whose expression increases with MIR181A1HGkd. TCF4 is a critical transcriptional factor in the Wnt/β-catenin pathway known to be essential for osteogenesis ^39^. One of the downstream SOX5 targets is TP53 which is upregulated in MIR181A1HG knockdown cells (all shown in Supplementary Fig. 5). The combined observations of MIR181A1HG binding upstream of SOX5, together with its subsequent downregulation after MIR181A1HG knockdown and the altered expression of SOX5 target genes suggest that MIR181A1HG knockdown downregulates SOX5 expression to impact osteogenesis.

## Conclusion

In this study we have identified a key lncRNA MIR181A1HG that contributes to early stages of MSC differentiation, which is essential for skeletal development, by regulating the expression of genes that are linked to both osteogenic and chondrogenic lineages. Depletion of MIR181A1HG during induction of MSC differentiation modulates extracellular matrix biosynthesis and dramatically increases ALP activity, reflecting early stages of differentiation of the osteo-chondroprogenitor phenotype. Further, lncRNA MIR181A1HG supports proliferative self-renewal of MSCs and is required for maintenance of genomic integrity and normal cell cycle progression. MIR181A1HG binds chromatin at both its own gene locus and at the SOX5 gene, and regulates SOX5 expression, thereby influencing its downstream targets. The network of genes controlled by MIR181A1HG defines novel regulatory pathways during MSC differentiation that can potentially be targeted for clinical conditions requiring new bone formation. It will be informative to explore in the future the extent to which MIR181A1HG controls regulation of the different types of bone tissues throughout the skeleton.

## Methods

### Cell culture and induction of MSC differentiation

The immortalized human mesenchymal stromal cell line (hMSC-hTERT20) was generously provided by Dr. Moustapha Kassem (University of Southern Denmark, Odense, Denmark)^40^. Cells were maintained and expanded in ascorbic acid-free alpha-MEM (Hyclone, Novato, CA, USA) supplemented with 16.5% fetal bovine serum, 2 mM L-glutamine, 100 U/ml of penicillin and 100 μg/ml streptomycin. Cells were maintained at 37 °C in a humidified 5% CO_2_ environment and media replaced every 2 to 3 days for the duration of all experiments.

Osteogenic differentiation was induced in complete alpha-MEM supplemented with 280 μM ascorbic acid, 10 mM beta-glycerophosphate, 10 nM dexamethasone and 100 ng/ml BMP2 (R&D Systems). Cells were maintained at 37 °C in a humidified 5% CO_2_ environment and media replaced every 2–3 days for the duration of all experiments. Differentiation was verified by the level of alkaline phosphatase (ALP) activity staining^41^. ALP-stained cultures were captured with a Leica dissecting microscope (M165FC) equipped with a Leica digital color camera (DFC310FX, Leica Microsystems Inc).

### Generation of knockdown cells

Stable cell line with knockdown of MIR181A1HG was generated using CRISPR interference. The pLV hU6-sgRNA hUbC-dCas9-KRAB-T2a-GFP plasmid was a gift from Charles Gersbach (Addgene plasmid #71,237; http://n2t.net/addgene:71237; RRID: Addgene_71237)^42^. hTERT20-hMSC cells were transduced with lentivirus to stably express sgRNA-dCas9-KRAB. MIR181A1HGkd cells contained an sgRNA (TACAGCCCATGTGAAAGACA) targeting the promoter region of MIR181A1HG, whereas the control cell line contained a non-targeting sgRNA (GGGAGGCAACTGAATCATGG). After infection, cells were expanded and sorted for GFP expression. Cells were passaged and resorted to ensure all cells contained dCas9-KRAB-GFP.

### RNA-sequencing

Total RNA was isolated from cells using Trizol and purified using the Direct-zol RNA Kit with DNaseI treatment (Zymo Research) according to the manufacturer's instructions. RNA quality and quantity were assessed using the RNA 6000 Nano Kit with the Agilent 2100 Bioanalyzer (Agilent Technologies). RNA quantity was further assessed by Qubit HS RNA assay (Thermo Fisher Scientific).

hTERT20-hMSC, control and MIR181A1HGkd libraries were built with the SMARTer Stranded Total RNA Sample Prep Kit—Hi Mammalian Kit (Takara) according to manufacturer’s protocol. Library quality was assessed by Bioanalyzer using DNA HS chip (Agilent). All RNA-Seq libraries were single-end sequenced (SE75) on a HiSeq-1500. Base calls and sequence reads were generated bcl2fastq software (version 1.8.4, Illumina). Six independent RNA-Seq libraries were prepared for each time point during differentiation of the hTERT20-hMSCs (n = 6) and 3 replicates were used for the MIR181A1HGkd and control cells (n = 3).

### Bioinformatics analysis

*RNA-Seq Analysis:* Data analysis was conducted using the Galaxy platform^43^ and RStudio^44^. Quality control analysis of fastq raw data was performed using FastQC^45^. Reads were aligned to reference genome (hg38) using STAR^46^, and reads were quantified using HTSeq-counts^47^ with Gencode annotation v37^48^. A cutoff value of 0.1 RPKM was used for gene expression. Differential expression analysis was performed with DESeq2^49^. For differential gene expression analyses, the cutoff for significant fold change was > 1.5, adjusted *p*-value < 0.05. Cells at two differentiation stages (day 7 and 14) were compared with undifferentiated cells (day 0) as well as to each other within each cell type (Control and MIR181A1HGkd).

Additionally, we examined the expression of MIR181A1HG in publicly available datasets of MSCs isolated from white adipose tissue (WAT), muscle (MUS) and bone marrow (BM)^28^. Datasets from twenty-one adult human tissues were downloaded from the ENCODE project^50^ and examined for MIR181A1HG expression as well (Supplementary Table 1).

*Hierarchical Clustering and Gene Ontology:* Genes were sorted into mRNAs and lncRNAs and clustering analysis of row normalized differentially expressed gene count data was performed for each group using K-means clustering and visualized using SeqSetVis^51^. mRNA groups with similar expression patterns were merged and Gene Ontology (GO) annotation analyses of gene sets were performed. GO analysis was derived from Gene Ontology (www. geneontology.org)^52,53^. GO Term enrichment was considered significant for all terms with *P* < 0.05. GO terms were consolidated using REVIGO^54^.

### ChIRP and ChIRP-seq

Chromatin Isolation by RNA Purification (ChIRP) experiments were performed as follows. 10^8 MSC-TERT20 cells were washed once by PBS and resuspended in 10 ml PBS. Cells were harvested by trypsin digestion, collected by centrifugation and initially crosslinked with 2 mM disuccinimidyl glutarate (DSG, Thermo Fisher) in PBS at room temperature (RT) for 30 min with mild agitation. Cells were then further crosslinked by addition of methanol-free formaldehyde (final concentration 3.7%) for 10 min and then quenched with excess glycine addition (250 mM) at RT for 5 min. Cells were collected by centrifugation and resuspended in fresh lysis buffer (100 mM Tris pH 7.0, 10 mM potassium acetate, 15 mM magnesium acetate, 1% NP-40, 1 mM DTT, 1 mM PMSF, 1 × Complete protease inhibitor (Roche), and 0.1 U/μl RNAse inhibitor Superase-in (Invitrogen) for 10 min on ice. The cell suspension was then homogenized (Dounce type A) and liberated nuclei collected by centrifugation (2500 g, 5 min). Pelleted nuclei were resuspended with nuclear lysis buffer (50 mM Tris pH 7.0, 10 mM EDTA, 1% SDS, 1 mM DTT, Protease Inhibitor and RNase Inhibitor) and incubated on ice for 10 min. Chromatin was sheared by sonication (Covaris E200) into 200–500 bp fragments and cleared by centrifugation for 10 min, and the resultant cleared supernatant moved to a new tube. For RNA hybridization, lysates were initially incubated with 100 nM biotin-conjugated LINC-specific probes (see Supplementary Table 2) in hybridization buffer (Final conc: 250 mM NaCl, 0.5% SDS, 50 mM Tris pH 7.0, 10 mM EDTA, 7.5% Formamide, 10 mM DTT, Protease Inhibitor and RNase Inhibitor) for 3 h at 39 °C with rotation. Streptavidin M280 beads (Invitrogen/ThermoFisher) were then added and incubated for an additional 3 h. After hybridization, bead complexes (that include magnetic beads: biotin-conjugated probes: crosslinked RNA: chromatin) were collected by neodymium magnet and washed 5 times with 0.1 X SSC wash buffer (0.1 X SSC, 1% SDS) at 42 °C with gentle agitation (ThermoMixer) for 5 min followed by magnetic collection. After the final wash, DNA was eluted by resuspending beads in DNA elution buffer (50 mM NaHCO3, 1% SDS, 200 mM NaCl with 100 μg/ml RNase A (Sigma-Aldrich) and 0.1U/μl RNase H (NEB) for 60 min at 37 °C with gentle agitation (ThermoMixer). Beads were then collected (neodymium magnet), supernatant removed, a second round of elution was performed, and the eluent from both steps was combined. Chromatin was then reverse-crosslinked by proteinase K treatment (0.2U/μl) at 65 °C for 60 min with gentle agitation (ThermoMixer) followed by purification using the MinElute PCR Purification Kit (QIAGEN). Eluted DNA was then used for library construction using the TruSeq ChIP library preparation kit (Illumina) following the manufacturer’s protocols and quantified using Qbit (Qiagen) and Bioanalyzer (Agilent). The resultant libraries were then subjected to qPCR, and high-throughput sequencing.

For qPCR analysis of ChIRP libraries, PCR primers were designed to specific genomic regions corresponding to gene exons or regulatory regions (e.g., enhancers, promoters)(see Supplementary Table 2). qPCR was carried out in reactions using 10 pg of library DNA using QuantiFast SYBR Green qPCR kit (Qiagen) using standard cycle parameters on Viia7 Real-time PCR thermocycler (ThermoFisher). Relative enrichment was calculated by normalizing Ct values to recovered input DNA (percent input) and then calculating fold enrichment compared to the control LINC (NEAT1).

### RNA in situ hybridization

RNA fluoroscence in situ hybridization (RNA FISH) was performed using RNAscope reagents, a HybEz oven, and custom probes targeting MIR181A1HG (Advanced Cell Diagnostics Bio-Techne), according to the manufacturer's protocols. Control assays were performed using a Homo sapiens PPIB as a positive control and an Escherichia coli dapB probe as a negative control. The nuclei were counterstained with DAPI. RNase A pretreatment was included to confirm probe hybridization to RNA. Images were obtained using a Zeiss LSM 510 META confocal microscope using a 63 × oil immersion objective.

### Growth assay

Cells were seeded in six-well plates at 5.2 × 10^3^ cells/cm^2^. Cells were trypsinized and counted over 4 days using a Countess Automated Cell Counter; 0 h is the time at plating (n = 3).

### Flow cytometry analysis

Cells were harvested by trypsinization and fixed in ice cold 75% ethanol for 30 min at 4 °C. For mitotic indexing, cells were incubated with Alexa Fluor 647 Rat anti-Histone H3 (pS28) (1:50; BD Biosciences 558,609) in permeabilization buffer for 30 min at room temperature in the dark. For mitotic indexing and cell-cycle analysis, cells were stained with propidium iodide (PI/RNase staining buffer, BD Biosciences: 550,825) for 15 min at room temperature in the dark. Flow cytometry was performed using an LSRII (BD Biosciences). Flowjo v10 (http://www.flowjo.com/) was used for analyses of cell cycle fractions using PI as well as to determine the percent of H3S28P-positive cells.

For analysis of S- phase fraction of cells and overall DNA content, BrdU (5-Bromo-2′-deoxy-uridine) incorporation assay using FACS was performed, cells were harvested at 90% confluency and stained with APC BrdU Flow Kit (BD Biosciences) with 7-AAD according to manufacturer’s protocol.

### BrdU incorporation assays (Immunofluorescence (IF))

BrdU incorporation was also assayed using IF to evaluate the fraction of cells in S phase using a 5-Bromo-2′-deoxy-uridine (BrdU) Labeling and Detection Kit according to the manufacturer’s protocol (Sigma-Roche). Briefly, cells were grown on coverslips to 50–70% confluence. BrdU (10 uM) was incorporated for 30 min at 37 °C, then cells were fixed with ethanol/50 mM glycine (pH 2.0) for 20 min at -20 °C. BrdU was detected by incubation with mouse IgG anti-BrdU primary antibody for 30 min at 37 °C (1:10) followed by Alexa Fluor 568 goat anti-mouse IgG for 30 min at 37 °C (1:500). Cells were counterstained with DAPI for 1 min and mounted with ProLong Gold Antifade (Thermo Fisher). Cells were imaged on a Zeiss AxioImager2 equipped with Hamamatsu CCD camera, and images were captured using Zen2012 software. Image analyses were performed using ImageJ (https://imagej.nih.gov/).

### Immunofluorescence (IF) microscopy

Cells were grown on coverslips until they reached 50–70% confluence. Cells were fixed in 3.7% formaldehyde, washed with PBS, permeabilized in 0.25% Triton X-100 in PBS, then rinsed with PBSA (0.5% bovine serum albumin (BSA) in PBS). Cells were stained with primary antibody for 45 min at 37 °C, followed by detection of antigen–antibody complexes using complementary fluorescently labeled secondary antibodies. Nuclei were counter-stained with DAPI for 1 min. Stained cells were mounted with ProLong Gold Antifade reagent.

Antibodies were used as described. To assess phosphorylation of histone H3 at the serine 28 residue, denoting mitotic cells, H3S28 antibody (1:500; EMD Millipore cat# 07–145) was used. To assess DNA double strand breaks and DNA damage, we stained for 53BP1 (1:500; SantaCruz clone H300) and pH2Ax Ser139 **(**1:500; EMD Millipore cat# 05–636 cloneJBW301). Appropriate Alexa fluor secondary antibodies were used.

53BP1 and pH2Ax specific staining were analyzed utilizing Perkin Elmer’s Volocity 6.3 Software. H3S28 specific staining was analyzed by quantifying the number of positively versus negatively stained cells.

### Statistical analyses

Statistical analyses were performed using GraphPad Prism v8.4.3.

## Supplementary Information

Supplementary Information 1.

Supplementary Information 2.

Supplementary Information 3.

## Data Availability

All datasets have been deposited in the Gene Expression Omnibus (GEO). Data for the hTERT20-hMSCs are under accession code GSE183931 and CRISPRi control and MIR181A1HGkd cells are under GSE184087.
