# Supplementary material for: LncMIR181A1HG is a novel chromatin-bound epigenetic suppressor of early stage osteogenic lineage commitment
Source: Sci Rep. 2022 May 11;12:7770. doi: 10.1038/s41598-022-11814-4 (PMC9095685; doi:10.1038/s41598-022-11814-4)
Supplement: Supplementary file 1 — Supplementary Information 1. [file 41598_2022_11814_MOESM1_ESM.pdf]

Title: LncMIR181A1HG is a novel chromatin-bound epigenetic suppressor of early stage osteogenic lineage commitment

Coralee E. Tye[1,2], Prachi N. Ghule[1,2], Jonathan A.R. Gordon[1,2], Fleur S. Kabala[1], Natalie A. Page[1], Michelle M. Falcone[1], Kirsten M. Tracy[1], Andre J. van Wijnen[1], Janet L. Stein[1,2], Jane B. Lian[1,2] and Gary S. Stein[1,2*]

Affiliation

[1]Department of Biochemistry and [2]University of Vermont Cancer Center, University of Vermont Larner College of Medicine, Burlington, VT, 05405, USA.

SUPPLEMENTAL FIGURES

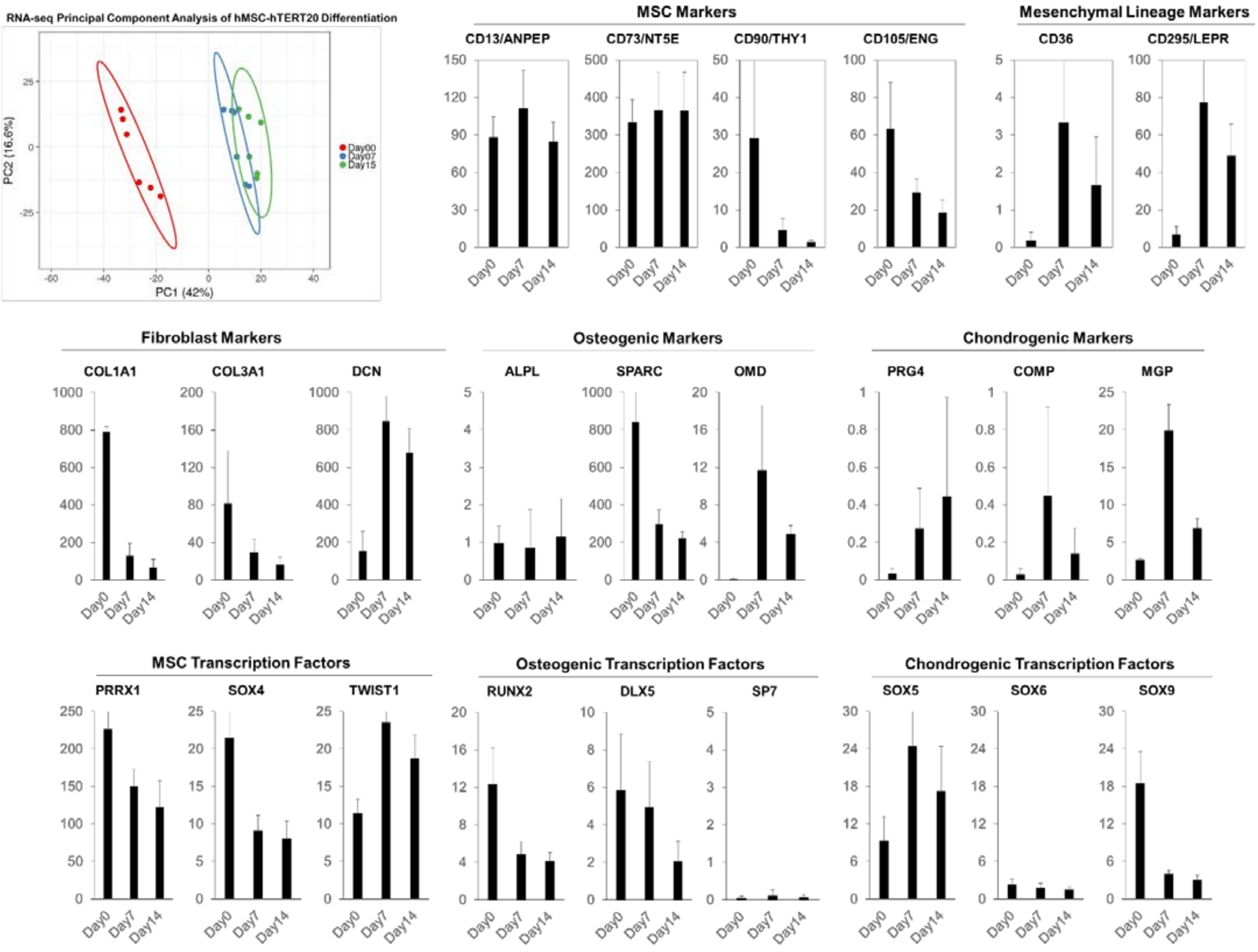

Supplementary Figure 1: RNA-Seq analysis during osteogenic differentiation of hMSC-hTERT20 cells. Top left panel: Principal Component Analysis (PCA) showing that gene expression of control samples (red; n=6) differs in the first dimension (PC1) from expression patterns of differentiated cells on Day 7 (blue; n=6) or Day 15 (green; n=6); the PCA image was generated using ClustVis after elimination of uninformative house keeping genes by selecting for genes (n=1,000) with the largest coefficient of variation (CV>0.7) and robust expression (normalized read value >1). Remaining panels: bar graphs of select groups of genes relevant to multi-lineage commitment of MSCs. MSC markers: classical CD cell surface markers used for establishing MSC identify. Mesenchymal Lineage Markers: CD markers known to be increased during adipogenic (CD36) or osteogenic (CD295) lineage commitment. Fibroblast, osteogenic and chondrogenic markers represent classical mRNAs that are reliable biomarkers for the indicated lineages. MSC, osteogenic and chondrogenic transcription factors were selected based on well-established regulatory roles in the indicated cell lineages.

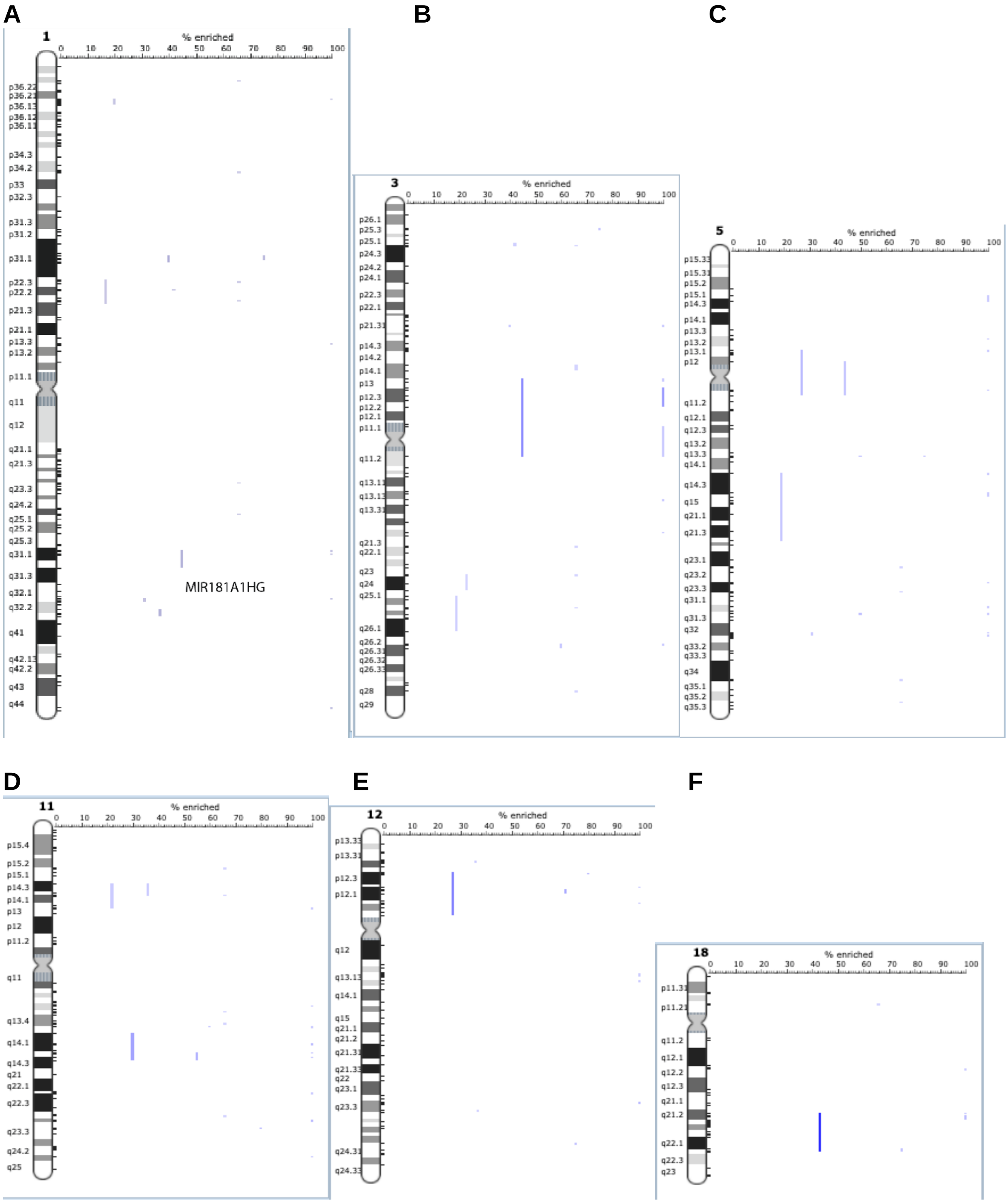

Supplementary Figure 2. Chromosome regions significantly enriched in genes differentially expressed after MIR181A1HGkd. Regions are plotted by the percentage of enrichment for (A) chromosome 1, (B) chromosome 3, (C) chromosome 5, (D) chromosome 11, (E) chromosome 12 and (F) chromosome 18.

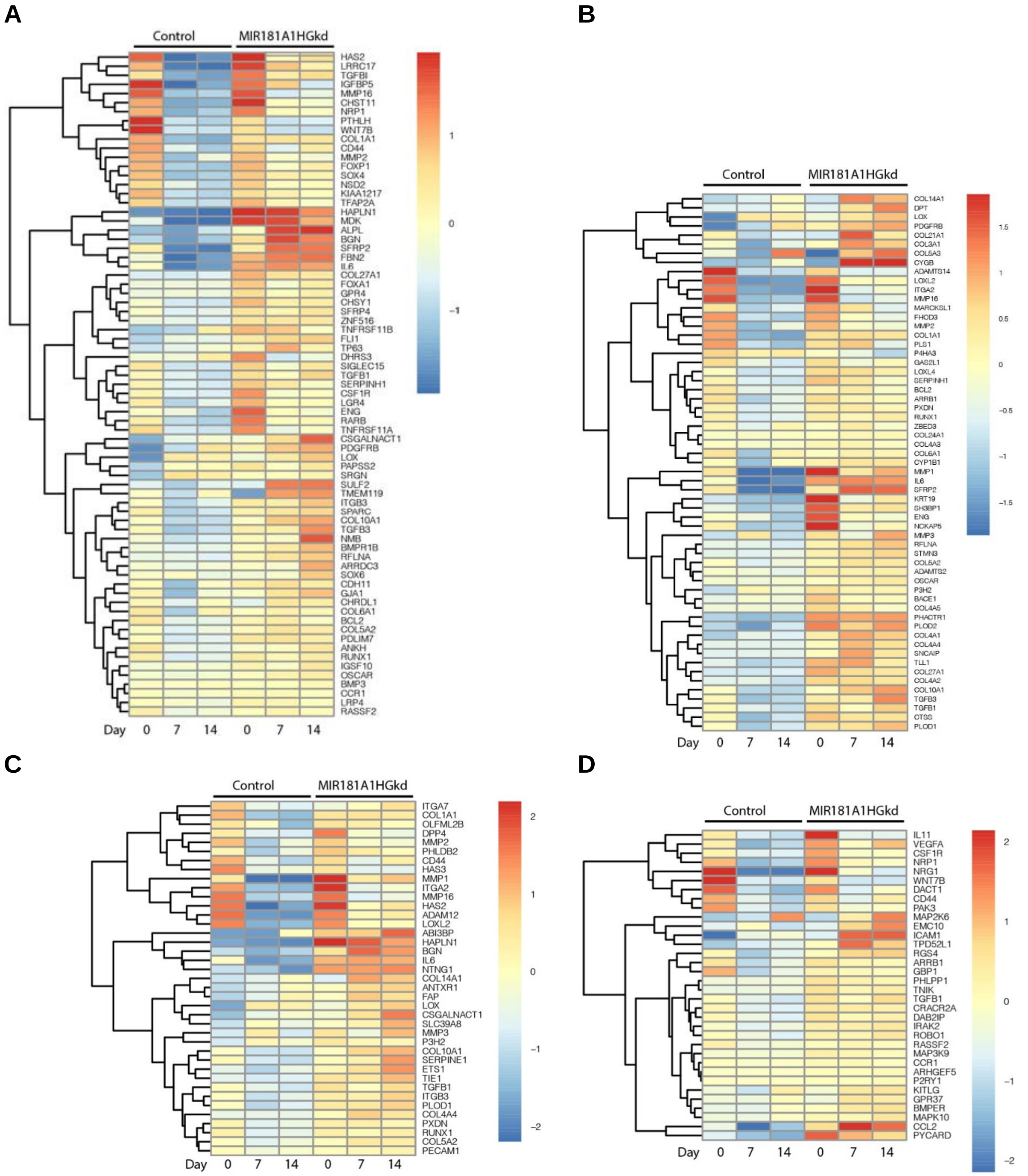
A
Control
MIR181A1HGkd
HAS2
LRRC17
TGFBI
IGFBP5
MMP16
CHST11
NRP1
PTHLH
WNT7B
COL1A1
CD44
MMP2
FOXP1
SOX4
NSD2
KIAA1217
TFAP2A
HAPLN1
MDK
ALPL
BGN
SFRP2
FBN2
IL6
COL27A1
FOXA1
GPR4
CHSY1
SFRP4
ZNF516
TNFRSF11B
FLI1
TP63
DHRS3
SIGLEC15
TGFB1
SERPINH1
CSF1R
LGR4
ENG
RARB
TNFRSF11A
CSGALNACT1
PDGFRB
LOX
PAPSS2
SRGN
SULF2
TMEM119
ITGB3
SPARC
COL10A1
TGFB3
NMB
BMPR1B
RFLNA
ARRDC3
SOX6
CDH11
GJA1
CHRDL1
COL6A1
BCL2
COL5A2
PDLIM7
ANKH
RUNX1
IGSF10
OSCAR
BMP3
CCR1
LRP4
RASSF2
1
0
−1
Day 0 7 14 0 7 14
B
Control
MIR181A1HGkd
COL14A1
DPT
LOX
PDGFRB
COL21A1
COL3A1
COL5A3
CYGB
ADAMTS14
LOXL2
ITGA2
MMP16
MARCKSL1
FHOD3
MMP2
COL1A1
PLS1
P4HA3
GAS2L1
LOXL4
SERPINH1
BCL2
ARRB1
PXDN
RUNX1
ZBED3
COL24A1
COL4A3
COL6A1
CYP1B1
MMP1
IL6
SFRP2
KRT19
SH3BP1
ENG
NCKAP5
MMP3
RFLNA
STMN3
COL5A2
ADAMTS2
OSCAR
P3H2
BACE1
COL4A5
PHACTR1
PLOD2
COL4A1
COL4A4
SNCAIP
TLL1
COL27A1
COL4A2
COL10A1
TGFB3
TGFB1
CTSS
PLOD1
1.5
1
0.5
0
−0.5
−1
−1.5
Day 0 7 14 0 7 14
C
Control
MIR181A1HGkd
ITGA7
COL1A1
OLFML2B
DPP4
MMP2
PHLDB2
CD44
HAS3
MMP1
ITGA2
MMP16
HAS2
ADAM12
LOXL2
ABI3BP
HAPLN1
BGN
IL6
NTNG1
COL14A1
ANTXR1
FAP
LOX
CSGALNACT1
SLC39A8
MMP3
P3H2
COL10A1
SERPINE1
ETS1
TIE1
TGFB1
ITGB3
PLOD1
COL4A4
PXDN
RUNX1
COL5A2
PECAM1
2
1
0
−1
−2
Day 0 7 14 0 7 14
D
Control
MIR181A1HGkd
IL11
VEGFA
CSF1R
NRP1
NRG1
WNT7B
DACT1
CD44
PAK3
MAP2K6
EMC10
ICAM1
TPD52L1
RGS4
ARRB1
GBP1
PHLPP1
TNIK
TGFB1
CRACR2A
DAB2IP
IRAK2
ROBO1
RASSF2
MAP3K9
CCR1
ARHGEF5
P2RY1
KITLG
GPR37
BMPER
MAPK10
CCL2
PYCARD
2
1
0
−1
−2
Day 0 7 14 0 7 14

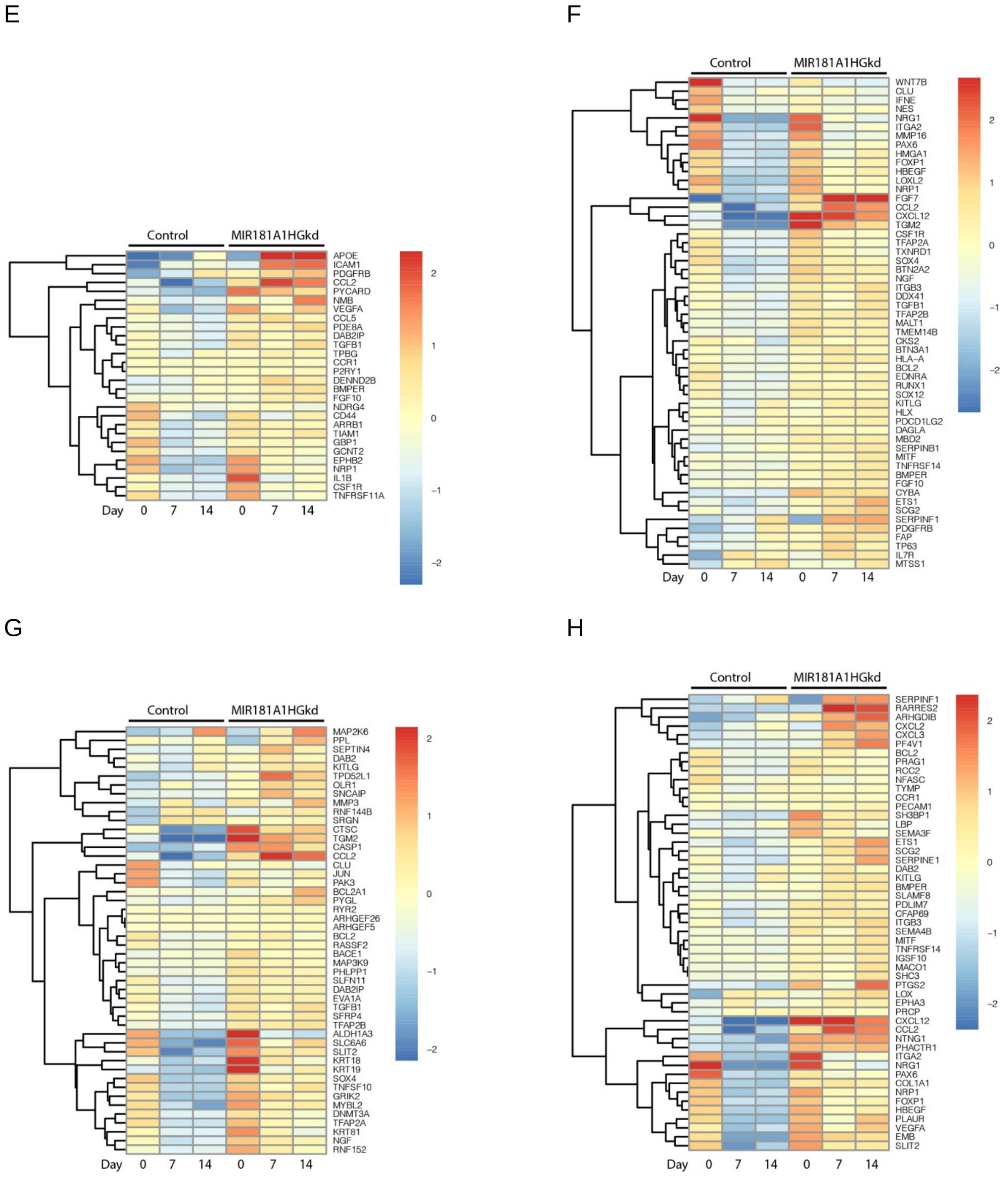

Supplementary Figure 3. Differentially Expressed mRNAs that are increased with MIR181A1HGkd MSCs during osteogenic differentiation and are associated with: (A) bone morphogenesis, bone development, skeletal system development, ossification, regulation of bone mineralization, regulation of bone remodeling; (B) collagen fibril organization, collagen-activated signaling and collagen metabolic process; (C) extracellular matrix organization; (D) positive regulation of MAPK signaling ; (E) regulation of ERK 1 and ERK2 cascade; (F) regulation of cell proliferation; (G) positive regulation of cell death; and (H) regulation of cell migration, regulation of cell motility, regulation of locomotion and taxis.

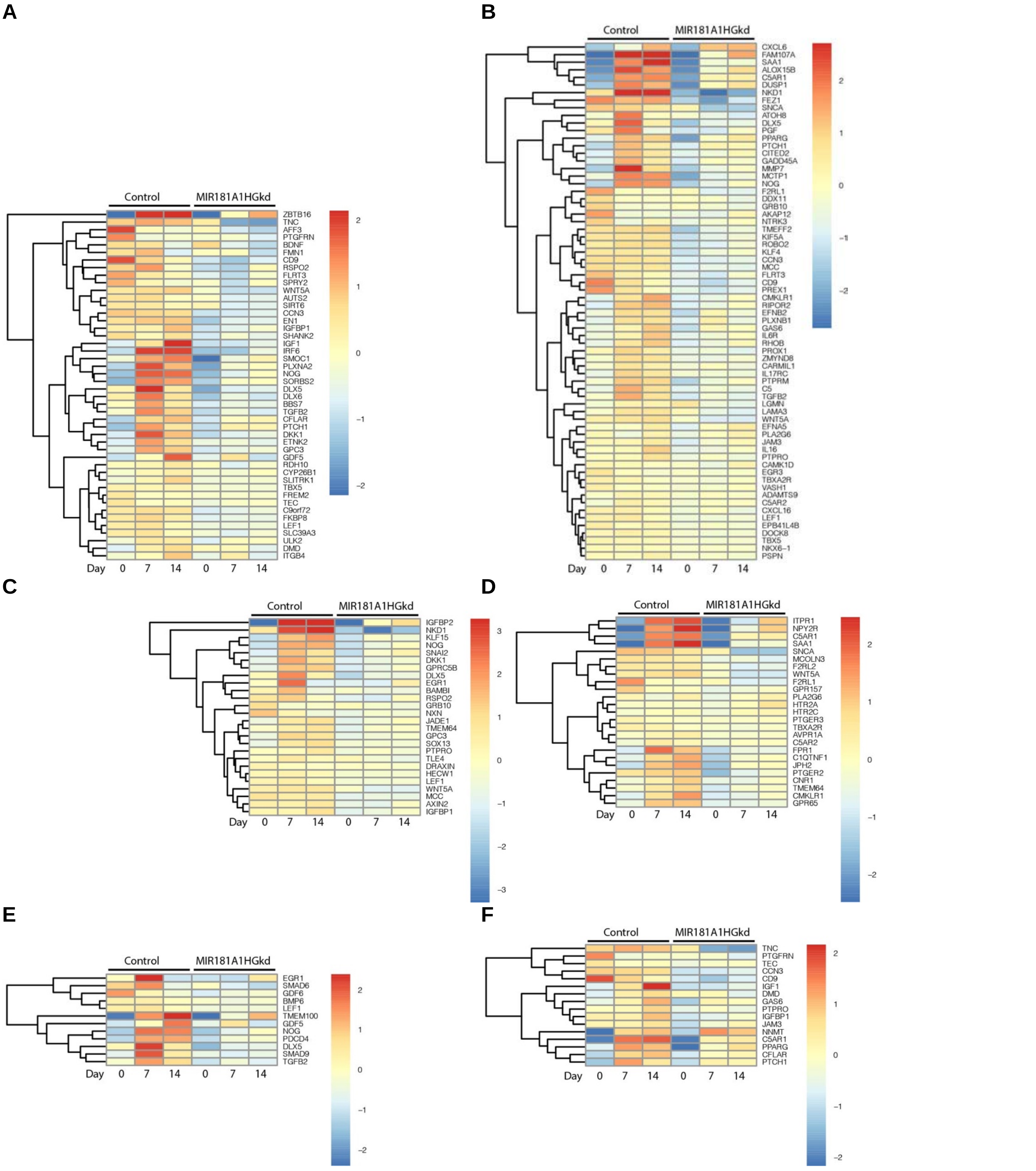

Supplementary Figure 4. Differentially Expressed mRNAs that decrease with MIR181A1HGkd MSCs during osteogenic differentiation and are associated with: (A) hindlimb morphogenesis, embryonic limb morphogenesis, and developmental growth; (B) regulation of cell migration, regulation of locomotion, regulation of chemotaxis and taxis; (C) regulation of WNT signaling pathway; (D) regulation of cytosolic calcium ion concentration; (E) BMP signaling pathway and response to BMP; and (F) regeneration.

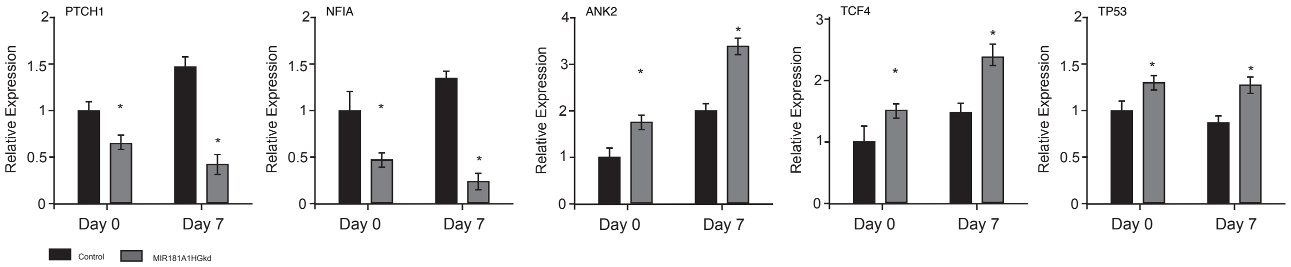

Supplementary Figure 5. Gene expression levels of SOX5 related genes in hMSC-TERT20 cells or MIR181A1HG knockdown cells. Real-Time PCR was used to assess expression levels of selected mRNAs. Total RNA was extracted from cultured cells using Trizol reagent. Total RNA (1μg) was used for cDNA synthesis. Quantitative PCR amplification was performed using the Viia 7 Real-Time qPCR thermocycler (Applied Biosystems/ThermoFisher) and SYBR™ Select Master Mix (ThermoFisher). For relative gene expression, GAPDH and HPRT1 were used for normalization. Data were calculated using the comparative Ct method (2–ΔΔ Ct) and expressed as fold change compared to day 0 controls. Gene-specific primer sequences are listed in Supplemental Table 2. RNA was isolated from 3 independent replicates and qPCR was performed with 3 technical replicates. Error bars represent S.D. between biological replicates, and statistical significance was determined by Student's t-test versus matched control (*P < 0.05).
